# A tool to support meaningful person-centred activity for clients with dementia – a Delphi study

**DOI:** 10.1186/s12912-015-0060-3

**Published:** 2015-03-06

**Authors:** Barbara Lloyd, Christine Stirling

**Affiliations:** Private Bag 135, School of Health Sciences, University of Tasmania, Hobart, 7000 TAS Australia

**Keywords:** Dementia, Person-centered care, Needs-assessment, Patient-care planning, Living alone, Identity

## Abstract

**Background:**

This paper reports on a study to validate the concept of the ‘Activity Support Tool’ that aimed to assist dementia service workers to identify and act upon the support needs of people with dementia living alone, in line with the person-centred ideal.

**Methods:**

The tool was part of a two-stage exploratory qualitative study, which used interview and observational data from seven people with dementia living alone. Findings highlighted that people with dementia use objects and spaces within their homes to maintain or re-enact identities from the past. Thematic results from interviews were translated into a tool, with construct validation using the Delphi technique. Eighteen expert health professionals received round one of the questionnaire and six participants completed round three. The first round directed our focus towards operationalizing the person-centred ideal of dementia care.

**Results:**

The tool was considered by almost all advisory panel members to be a potentially valuable resource for helping to address impediments to integrated, effective and person-centred dementia care. Specific strengths identified were simplicity, person-centeredness and applicability across service settings. Issues of concern included practicability, risk management, gender stereotyping and terminology. The results support the findings of previous research into the intuitive and ethical appeal, but problematic applicability, of person-centred dementia services.

**Conclusion:**

Health professionals with a range of service-related expertise found the concept of person-centred care compelling, but required tangible, enduring structures to translate the ideal into practical action. The tool now requires further research to test its usefulness in practice.

**Electronic supplementary material:**

The online version of this article (doi:10.1186/s12912-015-0060-3) contains supplementary material, which is available to authorized users.

## Background

Dementia is a disabling and ultimately fatal condition characterized by progressive deterioration of mental and physical functioning. Around 35.6 million people worldwide have dementia and this number is predicted to more than treble by 2050 [1]. The condition can impose devastating psychosocial burdens on people with dementia and their carers [2,3]. The financial cost of health and social care, combined with loss of income for the person with dementia and their carers, is estimated to be more than US$604 billion per annum [1]. Complicating the issue are the rising numbers of people with dementia living in single person households [4]. Although living alone can be a liberating experience for mainstream populations [5] it is a considerably more problematic option for those with dementia, who experience progressive memory loss, disorientation, decreasing capacity for self-care and social isolation [1].

Despite the risks, continued residence in the family home, rather than admission to residential care, is the preferred option for many people with dementia who live alone [6]. It is also a policy objective to reduce costs and relieve the crisis of unavailability of appropriate institutional care for people with dementia [7]. These facts have pressing implications for the delivery of productive and cost-effective health and social services. In the early to moderate stages of dementia, services can enable those with dementia to remain in their own homes for longer periods and with enhanced quality of life [8]. However those who live alone will eventually require institutional care if they do not die of other causes before the disease is advanced, or do not have a full-time, live-in carer. In such cases, service objectives must be adapted over time, with the goal of supporting residence in the private home eventually being superseded by that of a smooth transition to residential care. As with other chronic illness contexts, dementia trajectories require sensitive, ongoing support and guidance for people with dementia and those who care for them, as they prepare for crisis events and manage change [9]. As many people with late-stage dementia are frail, of advanced age and have co-morbidities [10], providing quality transitional care at the appropriate time constitutes a particular challenge for health and social care [11].

Well-intentioned interventions may not meet the more complex needs of clients, however, and may have unintended negative consequences. A focus on the human dimension can be lost amid large-scale institutional priorities [12]. Insufficient attention to the psychosocial aspects of service use can threaten identities that have already been compromised by the disease [13]. Uncoordinated services within and across aged care sectors can be wasteful for funders and confusing for users [14,15]. Insufficient time and/or expertise can incline service workers towards prioritising the reduction of risk over supporting the independence of those with dementia [16]. At worst, the human rights of dementia clients may be breached in the context of service provision [17]. Responses that fixate on dementia symptoms, while overlooking the distinctive personality, preferences and interests of the individual with the disease, can undermine the very personhood of people with dementia [18].

Disease-focused approaches to dementia care have been widely superseded by an ideology of ‘person-centred care’, which prioritizes the remaining strengths and capacities, subjective experiences and personal goals of individual care recipients [18-20]. Knowing ‘what matters’ to each client, and the possibilities that exist within particular environments, are crucial components of person-focused models of care [21,22]. Person-centred dementia care practices can benefit both recipients and providers of health-related services, by reducing behavioural and psychological symptoms of dementia [23], and promoting continuity of identity and a sense of normality [20,24]. Services that generate positive client affect also benefit carers, who report experiencing a considerable reduction of stress when dementia services are accepted and enjoyed by care recipients [14]. Although the concept of person-centred care has been critiqued for its abstraction and imprecision [19,20] and for insufficient attention to human relationships [25], embodiment [26] and cultural diversity [27], the core notion of responding to clients as unique and worthy individuals has retained its ethical and intuitive appeal across service fields.

Venturato, Moyle & Steel [28], however, point to a disconnection between the rhetoric of person centred dementia care and the reality that practice continues to be organized around system imperatives of tasks, routines, rules and regulations. In part, they argue, this is due to the complex and sometimes competing nature of notions such as ‘quality of care’ and ‘quality of life’ and of the requirements and demands of diverse organizations. In essence, policies advocating more person-centred services are not easily translated into change on the ground [17,19]. Particular organizations may be inflexible, lacking appropriate structures to encourage reflection and open communication between management and other workers [29]. The capacity of ‘frontline workers’ [30] to attend to the more subtle psychosocial needs of dementia clients can be constrained by factors such as insufficient empowerment of clients, stressful and sometimes competing work demands and administrative pressures, diverse organizational cultures and inadequate training opportunities for staff [14,31,32]. Reflecting on the mismatch of rhetoric and reality in service fields, Brooker ([33] p11) comments that ‘Many of us live with the uneasy knowledge … that …the lived experience of care for people with dementia … is anything but person-centred.’ The dissonance and emotional strain experienced by workers who aspire to deliver person-centred care, but are routinely prevented from doing so by structural constraints, can be thus be counterproductive for achieving desired outcomes [31].

Various initiatives have sought to ground the notion of person-centred care in ‘concrete, observable actions’ ([23] p.34) by providing tangible enabling structures for service workers. Most pertinent of these for the purposes of this paper is the development of documentary tools such as patient records and care plans. The use of documentation is ubiquitous in health care and service settings, as health care services come under increasing pressure to improve and record the quality of their service provision. Documents that ‘map the patient’s journey’ throughout the disease trajectory can assist providers to understand the impact of their services on the lived experiences of clients, and to reflect on how those services might be optimized [34]. Many documents, such as care plans, aim to address non-medical aspects of patient profiles, such as recreational interests, preferences and positive pursuits. Williams et al. [31] note the need for this kind of documentation, whether it is spontaneously produced or formally regulated. ‘Individualised nursing care is supported by knowing the individual as a person’, they argue. ‘Often this involves relatives and friends providing information on the patient’s life history, including personality and preferences. A variety of different formats can be used to record this information, but one side of A4 can be just as meaningful’ ([31] p.15).

Despite the often valuable insights provided by carers and family members, however, proxies may not always be attuned to, or correctly interpret the perspectives of the person with dementia [35]. Direct contributions from dementia clients can offer more reliable information and provide the optimal means for others to understand the dementia experience [36]. Furthermore, information presented in a bureaucratically recognisable format will be more likely to be considered significant by professionals, and therefore to remain on the patient’s file.

This paper reports on an exploratory qualitative study investigating the quality of life priorities and service needs of people with dementia living alone without a main carer, and the translation of findings into a tool for service providers [37]. The ‘Activity support for clients with dementia’ tool is designed to support frontline workers to translate the person-centred ideal into concrete, integrated practice across dementia care settings such as community services, residential aged care, occupational therapy and nursing, with the first point of contact being service workers who operate within private homes. These workers are in a privileged position to notice what people actually do within their domestic spaces in real time, rather than what they merely report in retrospect. When preserved on record, personalized interactions with the person with dementia can help health and social services professionals to support them throughout the spatial and temporal trajectory of the disease.

## Methods

This qualitative study was conducted in partnership with Advocacy Tasmania, a service providing health care decision and access support for people with dementia living alone. Ethics approval was granted by the University of Tasmania’s Social Science Committee. We used a two stage approach. In the first stage, interview and observational data were collected from people with dementia living alone without a proximate carer. Advocates contacted participants meeting the following criteria: currently receiving received Advocacy Tasmania services, a diagnosis of early to moderate dementia, living in a single person household in Tasmania, and identified by advocates as a person who might be interested in participating in this particular study. The advocate explained the purpose of the study and discussed the information sheet with each potential participant. If the individual indicated interest in participating, the researcher accompanied the advocate on a visit for the purposes of consent. Consent was verbal (audio-recorded) and ongoing, rather than written and obtained prior to commencement. There is increasing recognition that mainstream consent practices provide a barrier for people with dementia to participate in research, as they rely too greatly on cognitive capacity and can even be perceived as threatening [37,38].

We adopted a model of consent that engaged the remaining strengths of participants, rather than heightening their weakness, while ensuring their rights were protected with the professional assistance of advocates. Interviews were conducted in participants’ homes and took between 25 to 45 minutes each, depending on the capacity of the individual. The questions were open-ended and elicited subjective impressions and evaluations of psychosocial and structural aspects of the home environment.

Interviews asked about general life satisfaction but foregrounded residential satisfaction and assistance. These were transcribed and thematically analysed by the two authors. Findings highlighted that people with dementia use objects and spaces within their homes to maintain or re-enact identities from the past, and that the meanings of space and objects change as the person re-imagines their identity [36]. The findings also showed how the environment and ‘will to mobility’ had important implications for access to public spaces for people with dementia and that both these shifts require thoughtful and person-centred planning from service providers. An extended analysis of findings of these interviews is reported [36] separately, as the focus of this paper is the development of the tool, by means of the Delphi technique. The interview/observation analysis informed development of a tool: a two-page template, an instruction sheet, and four examples [see Additional file 1]. The tool facilitates identification of material and spatial aspects of the home that enable or constrain activities that are personally meaningful and rewarding for clients’ identities. Participants’ responses drew attention to an unmet need for a tool to assist service workers to operationalize the ideal of person-centred care, with a view to supporting those with dementia without a main carer, who wish to remain in their homes to do so for longer, and with enhanced life satisfaction.

In the second stage, we validated the concepts in the tool using the ‘Delphi technique’ [39,40]. The Delphi is an iterative method often utilized in health research for the purpose of identifying degrees of consensus on practice-related issues within a group of diverse, knowledgeable advisors [41]. An expert panel is convened to provide feedback on a particular object, issue or process, often by means of emailed questionnaires. Twelve organizations employing health professionals with expertise in dementia care in five Australian states were initially identified, using an Internet search engine. Representatives of these organizations were contacted by phone to ascertain their interest in participation in the study as an expert advisor. Only three professionals contacted in this manner expressed an interest in participation. The most common reasons for refusal were lack of time and a reluctance to commit to ongoing involvement in an extra, non-essential activity. Fifteen additional participants were subsequently recruited directly from organizations known to the researchers, or through professional contacts. A copy of the information sheet was sent to all who had expressed an interest, together with a formal invitation to participate. Consent was obtained by means of an email response to an emailed invitation. Our participants included three community nurses, two academic nursing educators, a clinical nursing educator, two aged care/nursing academics, a dementia research academic, an occupational therapist, a CALD (Culturally And Linguistically Diverse) case manager, a CALD aged care professional, a dementia services co-ordinator and a director of aged care services. The identity of panel members was known by the moderator, but not to other panel members.

The questionnaire sent to the panel addressed the wording, presentation and usefulness of the tool, the clarity of the instructions and the type of service workers in his or her professional field who might use the tool, at what point of service delivery it might be used and in what context. Over three ‘rounds’, the moderator summarized the group feedback and sent the summary, together with a new questionnaire and a modified version of the tool, to participants who had responded to the earlier round. This process is usually repeated in Delphi studies until an acceptable degree of consensus (at least 75% agreement) in responses has been obtained. The validation process was affected by a large drop in participation after the first round, despite reminders being sent and due dates extended. Of the eighteen professionals who had initially agreed to participate, thirteen completed and returned the Round 1 survey. No response or further communication was received from the remaining five panel members, who were accordingly excluded from the study. The Round 2 survey was completed and returned by only seven participants. Six completed and returned the third and final survey. This participation decline and difficulty in recruiting are two issues commonly encountered in applications of the Delphi technique [42]. Nevertheless, the enthusiasm and high level of consensus demonstrated in the first round of responses was an unexpected but welcome development, which directed our emphasis towards the valuable insights provided by participants into the complexities of delivering person-centred dementia services. We foreground the professional concerns, needs and priorities identified by the expert advisors in the following section.

## Results

Overall, our expert advisors judged the tool to be useful. Of the fourteen panel members who returned the first survey, twelve responded that it was easy to understand, visually appealing, user-friendly and had a useful purpose. Ten judged the examples to be helpful and relevant to their own field of practice. Two academic researchers indicated that they would not have an opportunity to utilize the tool ‘in the field’ and one felt the tool was superfluous, writing that it duplicated existing practice. Specific strengths of the tool identified by participants were simplicity, person-centredness and applicability across service settings.

### Benefits of the tool

#### Simplicity

There was strong support for the way the tool simplified the ‘observations of the obvious’ and guided the user to act on these observations. Participants’ responses highlighted the need for clearly presented aids to support service workers in delivering strengths-enhancing, person-centred dementia services in a systematic way. Respondents reflected on the need for ‘obvious’ procedures to be ‘spelled out’ in a way that directs users in the field to apply them in concrete ways in specific situations.

Co-ordinator, dementia services: It provides a simple structure for staff to follow in gleaning not just WHAT is important to a client, but HOW to improve the person’s experience from the service.

#### Person-centredness

Participants responded positively to the person-centred goal of the tool. Their responses indicated that it would help care workers to think of ways to prioritize meaning and respect for who the person is now and has been in the past, rather than focusing on limitations and losses, in their interactions with clients. They also commented on its potential to encourage service workers to carry out meaningful activities in one-on-one situations..

Occupational therapist: Anyone from an occupational therapy background … would see the clear benefit and therapeutic value of working with activities of significance to the client. The approach advocated acknowledges the client’s “personhood” and strives to enhance it through meaningful activity and engagement.

Nursing educator: The focus on maintaining capacity and strengths is wonderful and will lead to enhanced health outcomes … The tool will be very useful to help students focus on the capacity building goal of health living.

The tool was also judged to be of potential value in the educational setting, to help develop skills in person-centred care:

CALD aged care professional: It could be used during training around person-centred care and behaviour management, to support workers, case managers and bicultural workers.

#### Wide range of uses

Panel members identified a wide range of applicability for the tool, over and beyond the ‘living alone’ context originally envisaged. A service organization director welcomed the opportunity to document cumulative insights and ideas as the service worker comes to know the client better, while a nursing educator pointed to its potential to promote innovative and integrated services.

Nursing educator: The specific challenge of asking what the service can do to support the activity is a strength of the form. The [example] of ‘Tony’ demonstrates how a service can think outside of the usual service to meet the identified need through collaboration with other services.

One community nurse provided a detailed account of various contexts in which the tool might be usefully employed, while the remainder commented specifically on their own fields of practice or expertise. These included staff training and service delivery in community, respite and residential care settings. Panel members also noted that support workers, case managers, informal carers, nurses, and leisure and lifestyle professionals could find the tool useful.

Nursing educator: With modification, it could be useful in residential care environments to enable setting up of appropriate activity stations for residents. Carers, leisure and lifestyle staff, nursing staff, to identify leisure preferences of residents and facilitate the implementation of suitable activities in appropriate environments.

One expert advisor (a dementia services coordinator) remarked that the tool would “help to identify “best-fit” of members for the various groups and scheduling of meaningful activities”. Another participant agreed, but expressed reservations about a potential unintended outcome that might constrain rather than enhance the quality of care offered.

Dementia research academic: [The tool] may prevent the tendency to just have any person doing any activity just for the sake of occupying time [and] increases the likelihood of continued engagement in either individual or group pleasures. Caveat: If the only activities the person is offered, or supported to continue, are those that fit expressed preference … no ‘new’ experiences – involving novelty, mystery – will be offered.

Only one participant considered that the tool had limited applicability and that its focus on activities might not be appreciated by clients.

Case manager, CALD services: I would imagine that it is mainly used with clients who are socially isolated, don’t have major other health problems that need to be attended first and who would profit from diversional therapy. Some clients may get a lot of support from their family in this area and prefer the support workers to do something else.

### Potential difficulties with the tool

Panel members found the ideology of person-centred care compelling, but identified particular issues commonly encountered in dementia service settings that could undermine the utility of the tool for translating that ideology into practice. Despite their overall acceptance of the tool, some participants articulated concerns and reservations arising from their own experiences and observations. These included practicability, risk management, gender stereotyping and terminology.

#### Practicability

Two advisors considered that work and time pressures frequently experienced by frontline workers and administrative staff might compromise the value of the tool. One noted that ‘both time and communication skills are essential to this tool’s success’.

CALD aged care professional: I am not entirely opposed to this section. However it could create the perception of a lot of paperwork for the staff member to complete for each client. Also it would need to be clear how often the service would expect this part of the doc to be completed.

#### Risk management

While one panel member suggested that the issue was of sufficient importance to be specifically addressed in the tool, the majority indicated that they considered this a complex matter, which would be more appropriately discussed with management within a wider care-planning context.

Director, aged care services: Activities should be about encouraging activity. Managing any risk to a particular person undertaking an activity is a component of the service provider care/service delivery planning discussions with the staff, person and their representative.

Case manager, CALD services: In most cases, I would expect that staff completing the form would be suggesting services in line with their organization’s Occupational Health and Safety policies. If the suggestion did not meet these requirements, management could point this out and ask for suggestions to modify the activity. If you mention risk avoidance on the form, you may risk stifling creative suggestions!

#### Gender stereotyping

Some experts believed that the usefulness and appeal of the examples were compromised by gender stereotypes. The uneasiness expressed by the CALD professional quoted below demonstrates the discomfort that perceived threats to the personhood of a person with dementia can engender, even when reflecting upon supposedly hypothetical situations.

CALD aged care professional: [The example of Anne] is a lovely scenario in many ways: pets have been proven to alleviate isolation and would provide many opportunities to engage with this client. The scenario would equally apply to men, so is gender-neutral. … I did have some difficulty relating to the teddy bear example [‘Mary’]. I know that people with dementia often enjoy what is normally considered a child’s toy. Even so, it still brings up feelings of poignancy for me.

#### Terminology

The final issue of concern for some participants was the use of the term ‘dementia’ in the title of the tool. While all respondents were cognizant of, and sensitive to, the stigma endured by people with dementia and their families, opinions were mixed as to whether the issue needed to be addressed at all on a document of this nature. In the opinion of some experts, it was best dealt with by tactful avoidance.

Dementia services coordinator: Alas, the stigma of dementia in the present means that many clients with dementia do not seem to want to acknowledge that they have “dementia”. Some lack insight into their condition and become quite distressed if dementia is mentioned. So, if the client is going to see the form as it is being completed, I would remove ‘dementia’.

One participant remarked that spoken conversations were more sensitive than the printed word, and terminology could be decided during individual encounters. Others believed that open and consistent usage of the word ‘dementia’ could enhance communication between service workers and clients, and help to de-stigmatize the condition. A decision was made by the researchers to retain the word in the final version of the tool, in accordance with majority panel opinion and in line with the classification of dementia as a ‘preferred term’ by Alzheimer’s Australia (2009).

### Observations on the Delphi process

Obtaining overall consensus was not difficult, and involved only minor adjustments to the tool. However some individual differences remained unresolved. In round 3, for example, we had added a new section ‘Suggestions for practice’ based on round 2 feedback that a more structured context was required for discussion between frontline service workers and management. While one participant felt it would be too time consuming, others indicated that they found the additional section useful for facilitating discussions of future care planning.

Community nurse: I really like this extra section. As well as providing an opportunity to determine/address potential risks, I see it also as a learning tool for supervisors and care staff. Requiring thought about the intended outcome of these activity supports offers an opportunity for staff to evaluate the implementation of tools/processes.

The examples presented more challenges, as they often elicited more personalised responses.

In one instance, we had replaced an example of a former mechanic ‘John’, who regularly tinkered with machinery parts in his shed, in order to address the criticism of gender stereotyping,. However, another advisor wrote that this had been a useful, realistic and gender-appropriate example, and that it should be retained.

Nursing educator. I do not think the examples are improved with the removal of John. John’s example is the only obvious past employment linked example and for many men work and the expert skills gained through employment are important to their self image. The example was a strong example of how important life satisfaction through employment is for many men and how this remains important through out life.

## Discussion

The results of this study support the findings of previous research into the intuitive and ethical appeal, but problematic applicability, of person-centred dementia services. Professional advisors with a range of health-related expertise found the concept of person-centred care compelling, but their responses indicated that they lacked tangible, enduring structures to translate it into practical action. The Delphi technique validated the concept of the tool and that it addressed a fundamental aspect of person-centred care: knowing the things that matter to each individual [21]. The findings suggest the tool could facilitate translation of this information into concrete actions, such as devising meaningful activities for people with dementia without a main carer. This is a much needed step in care planning actions [23]. The tool was considered by almost all Delphi group respondents to be a potentially valuable resource for helping to address the impediments to integrated, effective and person-centred care in health and social services for people with dementia. Their responses also confirmed others [42] observations as to differing perspectives of dementia service professionals on the issue of risk management.

## Conclusion

Our Delphi study findings have particular implications for improved practice. The first is the general observation that ethically sound concepts need appropriate, user-friendly structures to enable them to be translated into specific activities. The second is that certain client and service provider needs may be recognized subjectively and even articulated, but remain structurally unaddressed. The tool we have developed is simple in form and content, and draws upon knowledge that may be considered obvious by many professionals. Nevertheless, we have exposed an unaddressed need for such knowledge to be codified in a standardized format, which can remain on file to be accessed by diverse professionals across space and time. The third is the recognition that, although all people with dementia are different, individualized care need not be too resource-consuming. The observations and reflections of the Delphi group respondents in this project indicate that the basic tool ‘Activity support for clients with dementia’ could constitute a valuable addition to the professional resources of dementia workers and support enhanced quality of care in a variety of dementia service contexts.

This study has limitations. The purpose of the study must be kept in mind, which was to validate the concept of the tool. The small purposive samples used for the baseline interview data means the initial results are not generalizable and were theoretical in nature. The results from the Delphi Technique are dependent on the expertise and honesty of the group respondents and only validate the concept of the tool. Our group consisted of relevant experts in the field and the anonymity would have encouraged frank responses. However the tool now needs to be tested in practice and further research to validate its usefulness will be necessary.

## Additional file

Additional file 1:
**Activity support for clients with dementia.**
