# Supplementary material for: A tool to support meaningful person-centred activity for clients with dementia – a Delphi study
Source: BMC Nurs. 2015 Mar 6;14:10. doi: 10.1186/s12912-015-0060-3 (PMC4357087; doi:10.1186/s12912-015-0060-3)
Supplement: Additional file 1: — Activity support for clients with dementia. [file 12912_2015_60_MOESM1_ESM.pdf]

Activity support for clients with dementia:

The purpose of the tool

This tool provides a way for service providers to put the ideal of 'person centred care' into practice. It will help you to create more detailed and personalised care plans, by identifying interests and activities that are important and rewarding for individual clients.

Completed versions of the tool will remain on the client's file as a lasting record as dementia progresses. This will enable your organisation, as well as future service providers, to provide ongoing support that fits with the client's personality and preferences, even when the client is no longer able to communicate clearly. This is important, as carers and family members can be mistaken about the preferences and priorities of people with dementia.

Instructions for use

Step 1. During conversations with clients, make a note of any topic or activity that they do or mention often and seem to enjoy. This will be something that has been important to them in their life and continues to be an important part of how they see themselves.

Step 2. Confirm your impression with the client, saying something like "It seems to me that …. is very important to you. Is that right? Could you tell me more about that?"

Step 3. In a few words, describe this part of their life in the box marked: **'An important part of this client's life is …'**

Step 4. Observe what the client does that shows that this is important to him or her. Write this in the box marked: **What does he/she do that shows that this is important?**

Step 5. Observe the kinds of objects or things that clients use when they are doing this activity. For example, if the client enjoys painting as a recreational activity, write 'paint, paper, brushes,' in the box marked: **What thing(s) does he/she need to do this?**

Step 6. Through observing and talking to the client, decide whether these activities need to happen in the exact place the client now uses, or whether they could happen in a similar kind of place. For example, if the client enjoys cooking, is there something about his or her current kitchen that is very important, or would any kitchen serve the purpose just as well?

Step 7. Go to the box marked: **Where does this need to be done?** If they must use this exact place, then tick the box 'This particular place' and write what it is (e.g. 'this kitchen'). If they could do what is important to them in a similar place if the right things were provided, tick the box 'This kind of place' and write what it is (e.g. a kitchen).

Step 8. Now think of a service your organisation could provide, that could enable the client to keep doing this activity or to do it in a way that would be more personally satisfying. Write the name of these activities or services in the box marked: **What can my service do to support this activity?**

Step 9. With the aid of the completed form, discuss your ideas with your supervisor or service coordinator. Record the results of your discussion on the form 'Activity support for clients with dementia: suggestions for practice' and attach both forms to the client's file.

Activity Support for Clients with Dementia

Name of Client ....

▶ An important part of this client's life is

....

....

▶ What does he/she do that shows that this is important?

....

....

▶ What thing(s) does he/she need to do this?

....

....

▶ Where does this need to be done?

This particular place: ☐ ....

This kind of space: ☐ ....

▶ What can my service do to support this activity?

....

....

▶ Service Provider

Name: ....

Signature: .... Date: ....

Barbara T. Lloyd & Christine M. Stirling, 2012

Activity Support for Clients with Dementia: Suggestions for practice

▶ Proposed service or activity:

....

....

▶ Benefit/s to the client:

....

....

▶ Management of risk/s to client (if any):

....

....

▶ Management of risk/s to service provider (if any):

....

....

▶ Intended outcome/s for client:

....

....

▶ Intended outcome/s for service provider:

....

....

....	....
Name and role of service provider	Name of supervisor
....	....
Signature	Signature

Example One:

A client is widowed and has one son, who lives in the United States. She is very attached to her two dogs, Rover and Ginger, as she has had them since they were puppies. Rover and Ginger are free to come and go in her house and garden and they sleep in her room at night. She calls them her ‘best friends’ and says that having them around makes her feel safe.

The important part of her life would be her dogs. The activity you notice would be talking to her dogs and looking after them. The things she would need to do this would be her dogs and grooming equipment. The places she needs would be both ‘this particular place’ (for her own dogs) and ‘this kind of place’ (anywhere she can be with other dogs).

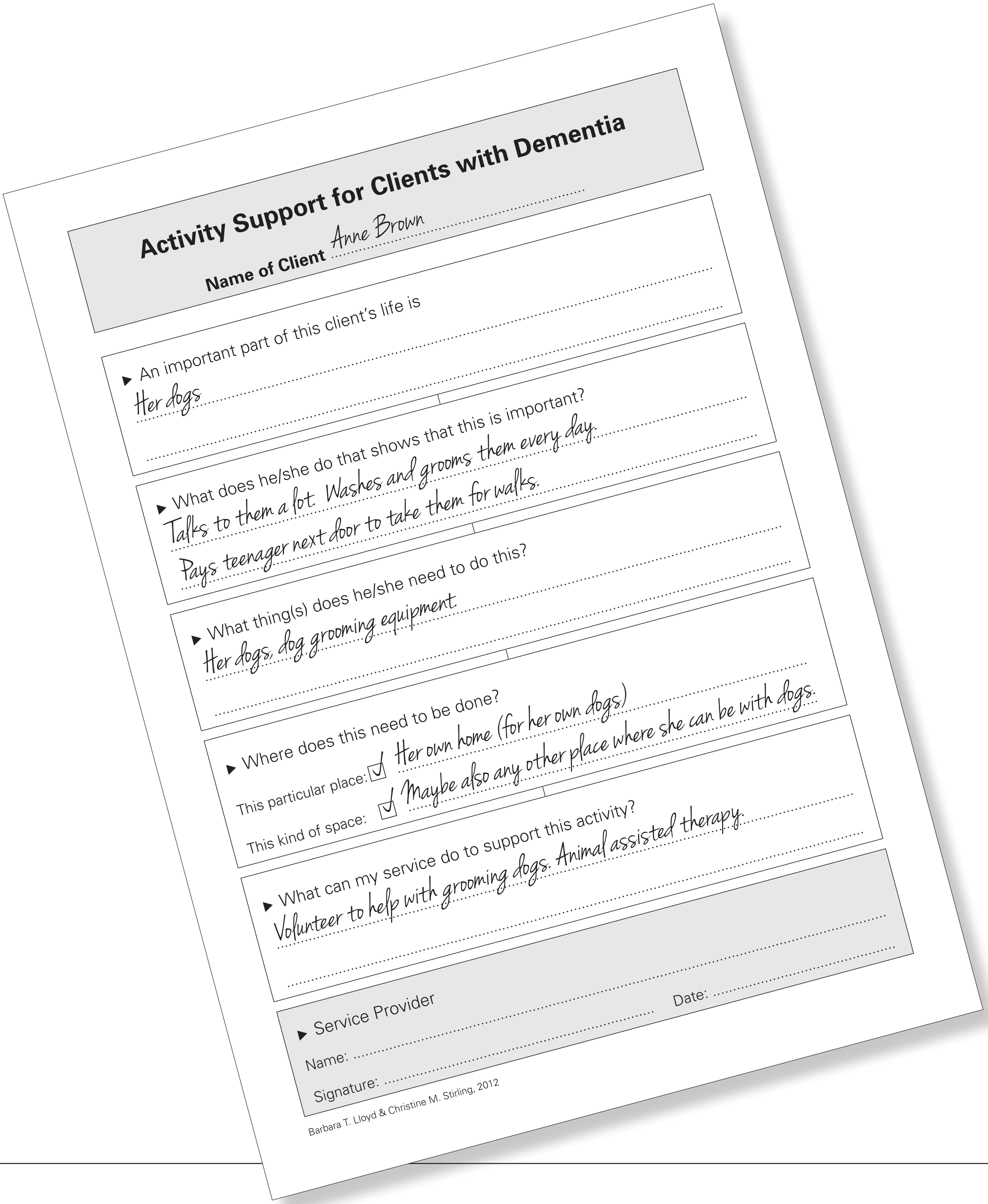

Activity Support for Clients with Dementia

Name of Client Anne Brown

▸ An important part of this client’s life is

Her dogs

▸ What does he/she do that shows that this is important?

Talks to them a lot. Washes and grooms them every day.

Pays teenager next door to take them for walks.

▸ What thing(s) does he/she need to do this?

Her dogs, dog grooming equipment.

▸ Where does this need to be done?

This particular place: ☑ Her own home (for her own dogs)

This kind of space: ☑ Maybe also any other place where she can be with dogs.

▸ What can my service do to support this activity?

Volunteer to help with grooming dogs. Animal assisted therapy.

▸ Service Provider

Name: .... Date: ....

Signature: ....

Barbara T. Lloyd & Christine M. Stirling, 2012

Example Two:

A client talks enthusiastically about his hobby of collecting and operating model trains. He spends several hours a day in his ‘train room’. This is a room he has dedicated to his hobby. It has pictures of trains on the walls and is lined with cabinets containing train memorabilia. There is a large table in the centre, permanently set up with model trains, tracks and scenery.

An important part of his life would be ‘His train hobby’. The activity you notice would be ‘doing things with model trains’. The things he would need to do this would be his model trains and scenery. The place he needs would be a particular place, i.e. the room he has set up especially for this activity.

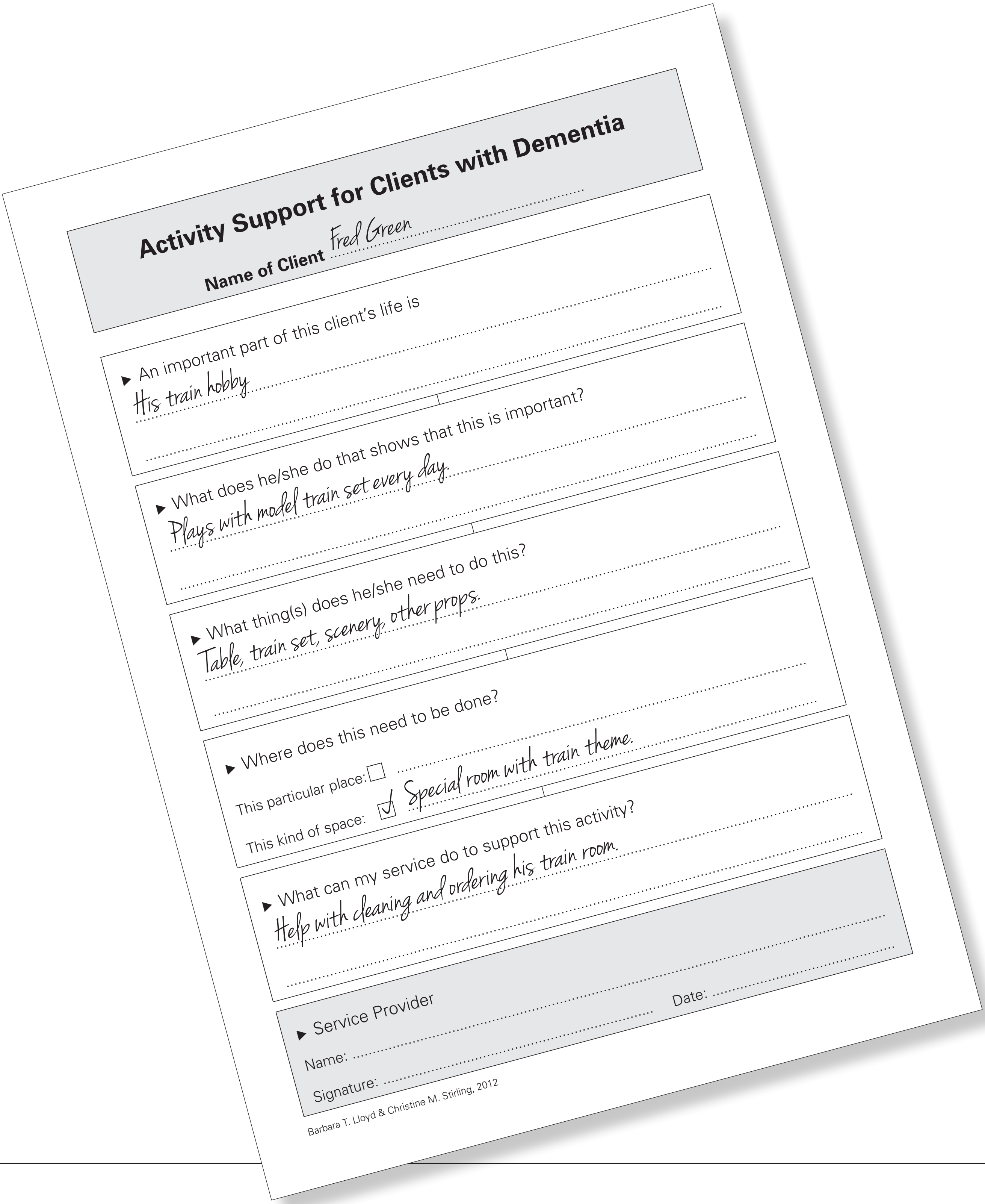

Activity Support for Clients with Dementia

Name of Client Fred Green

▸ An important part of this client’s life is

His train hobby

▸ What does he/she do that shows that this is important?

Plays with model train set every day.

▸ What thing(s) does he/she need to do this?

Table, train set, scenery, other props.

▸ Where does this need to be done?

This particular place: ☐

This kind of space: ☑ Special room with train theme.

▸ What can my service do to support this activity?

Help with cleaning and ordering his train room.

▸ Service Provider

Date:

Name:

Signature:

Barbara T. Lloyd & Christine M. Stirling, 2012

Example Three:

A client has never married and has no children. She frequently reminisces about her own happy childhood. Her furniture is covered with framed photographs of herself as a child, alone or with her loving parents. She has dolls and teddy bears on display in her bedroom and lounge room. Some are from her own childhood, while others have been more recently acquired. She often talks about how she came to have them and calls them her 'companions'. From time to time, she talks to them in a joking way, as though they were people.

An important part of her life would be 'Her childhood'. The things she would need to do this would be dolls, bears and photographs. The place she needs would be a kind of place, i.e. any place where she could act out her relationships with these things.

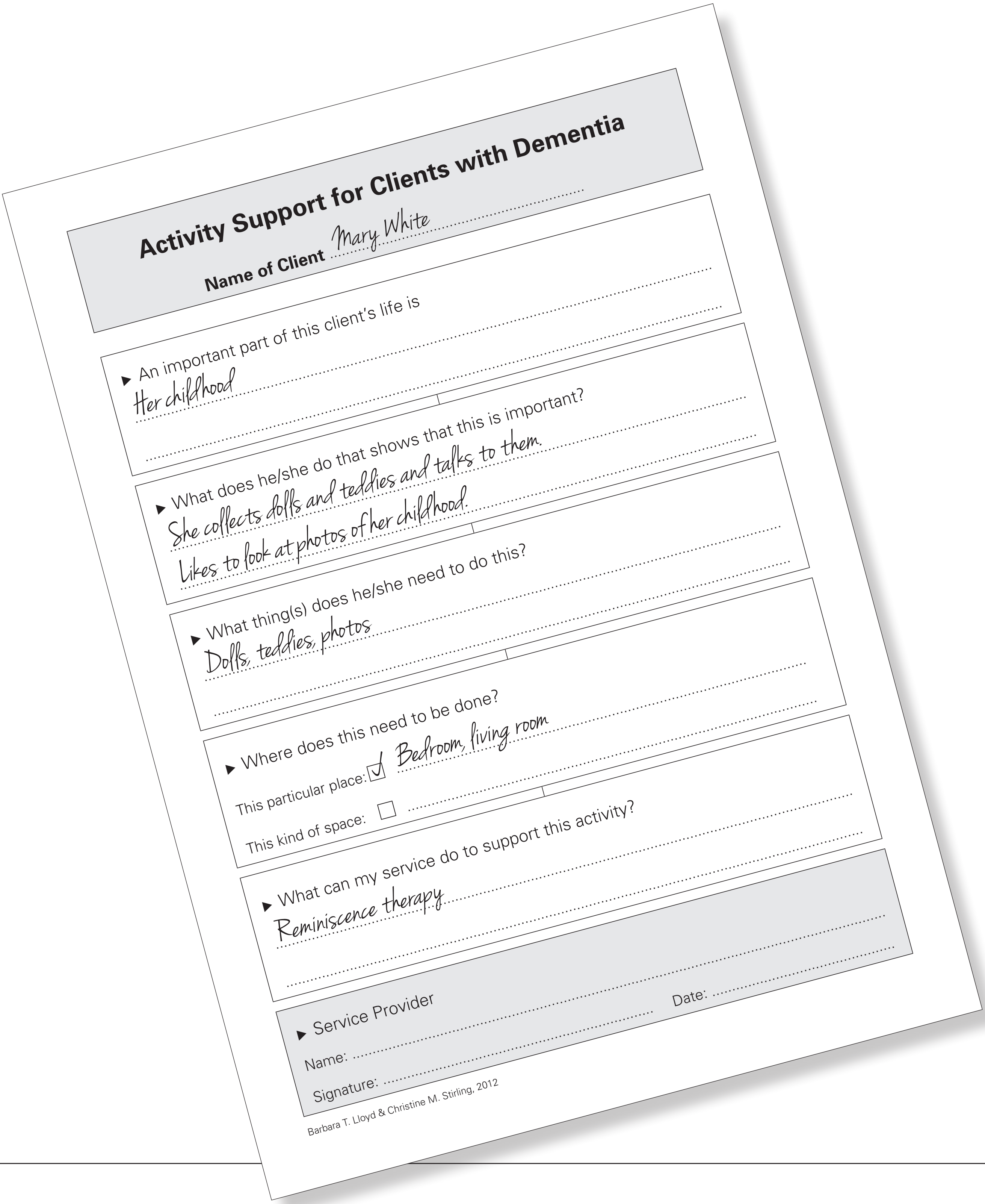

Activity Support for Clients with Dementia

Name of Client Mary White

▸ An important part of this client's life is

Her childhood

▸ What does he/she do that shows that this is important?

She collects dolls and teddies and talks to them.

Likes to look at photos of her childhood.

▸ What thing(s) does he/she need to do this?

Dolls, teddies, photos

▸ Where does this need to be done?

This particular place: ☑ Bedroom, living room

This kind of space: ☐

▸ What can my service do to support this activity?

Reminiscence therapy

▸ Service Provider

Name:

Signature:

Date:

Barbara T. Lloyd & Christine M. Stirling, 2012

Example Four:

Tony migrated to Australia from Italy forty years ago. He is very proud of his Italian-style garden, which has fruit trees and a greenhouse. He often remarks that the smell of the lemon tree takes him back 50 years. Lately he has begun to speak in Italian to people who don't understand the language.

An important part of his life would be 'His Italian garden'. The things he would need to do this would be a garden and tools. The place he needs would be an 'Italian style garden'.

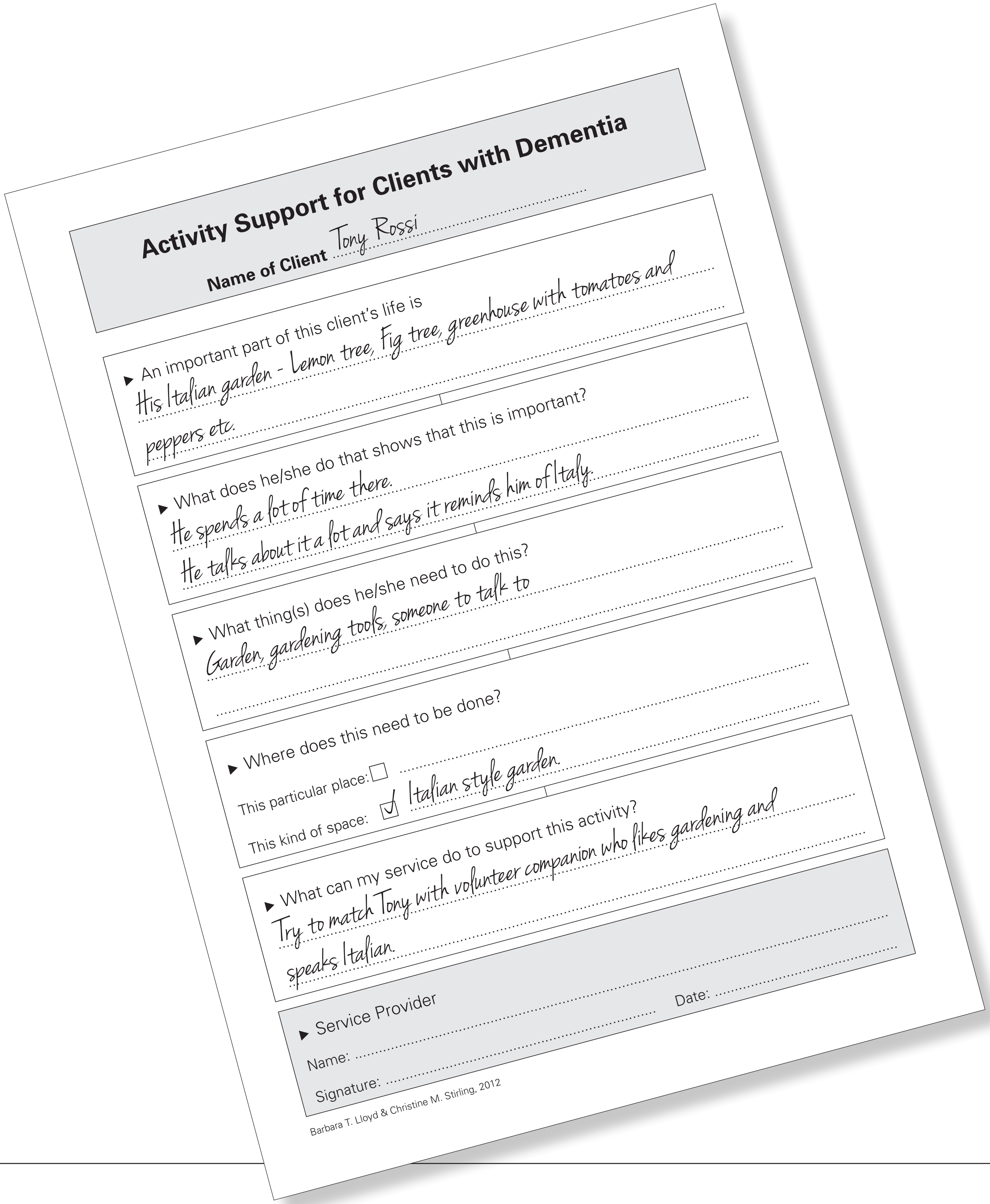

Activity Support for Clients with Dementia

Name of Client Tony Rossi

▸ An important part of this client's life is
His Italian garden - Lemon tree, Fig tree, greenhouse with tomatoes and peppers etc.

▸ What does he/she do that shows that this is important?
He spends a lot of time there.
He talks about it a lot and says it reminds him of Italy.

▸ What thing(s) does he/she need to do this?
Garden, gardening tools, someone to talk to

▸ Where does this need to be done?
This particular place: ☐
This kind of space: ☑ Italian style garden.

▸ What can my service do to support this activity?
Try to match Tony with volunteer companion who likes gardening and speaks Italian.

▸ Service Provider
Name:
Signature:
Date:

Barbara T. Lloyd & Christine M. Stirling, 2012
